# Genome-wide characterization of the *CPA* gene family in potato and a preliminary functional analysis of its role in NaCl tolerance

**DOI:** 10.1186/s12864-024-10000-2

**Published:** 2024-02-05

**Authors:** Jintao Liu, Dianjue Li, Jing Wang, Qian Wang, Xiao Guo, Qi Fu, Philip Kear, Guangtao Zhu, Xiaohui Yang

**Affiliations:** 1 Key Lab for Potato Biology in Universities of Yunnan, School of Life Sciences, Yunnan Normal University, Kunming, 650500 China; 2 Southwest United Graduate School, Kunming, 650500 China; 3 Institute of Vegetables, Shandong Academy of Agricultural Sciences/Molecular Biology Key Laboratory of Shandong Facility Vegetable/National Vegetable Improvement Center Shandong Sub-Center, Jinan, 250100 China; 4 International Potato Center (CIP), CIP China Center for Asia Pacific, Beijing, 100081 China

**Keywords:** Potato, CPA, NaCl stress, Gene expression, Ion transporter

## Abstract

**Background:**

The cation/proton antiporter (CPA) superfamily plays a crucial role in regulating ion homeostasis and pH in plant cells, contributing to stress resistance. However, in potato (*Solanum tuberosum* L.), systematic identification and analysis of *CPA* genes are lacking.

**Results:**

A total of 33 *StCPA* members were identified and classified into StNHX (*n* = 7), StKEA (*n* = 6), and StCHX (*n* = 20) subfamilies. StCHX owned the highest number of conserved motifs, followed by StKEA and StNHX. The StNHX and StKEA subfamilies owned more exons than StCHX. NaCl stress induced the differentially expression of 19 genes in roots or leaves, among which *StCHX14* and *StCHX16* were specifically induced in leaves, while *StCHX2* and *StCHX19* were specifically expressed in the roots. A total of 11 strongly responded genes were further verified by qPCR. Six *CPA* family members, *StNHX1*, *StNHX2*, *StNHX3*, *StNHX5*, *StNHX6* and *StCHX19*, were proved to transport Na^+^ through yeast complementation experiments.

**Conclusions:**

This study provides comprehensive insights into *StCPAs* and their response to NaCl stress, facilitating further functional characterization.

**Supplementary Information:**

The online version contains supplementary material available at 10.1186/s12864-024-10000-2.

## Background

Salt stress severely impacts plant growth and yield, affecting over 800 million hectares of land globally and hindering sustainable agriculture [1, 2]. NaCl, the most soluble and widely distributed soil salt, is absorbed by roots, accumulating in tissues and resulting in damage to plants [1, 3]. Throughout evolution, plants developed various mechanisms to regulate the intracellular ion balance and resist salt stress, relying on ion transporters in cell and organelle membranes [4].

The cation-proton antiporters (CPA) superfamily is a vital class of proteins in plants, facilitating the transport of Na^+^ as well as cations, such as K^+^ and Li^+^ [5–7]. The *CPA* superfamily comprises two broad categories, named CPA1 and CPA2 [8]. CPA1 includes the Na^+^/H^+^ exchanger family (NHX) [9, 10], while CPA2 includes the K^+^ efflux antiporter (KEA) and cation/H^+^ exchanger (CHX) family [5, 9]. *CPA* identification has been reported in various plants like *Arabidopsis thaliana* [11, 12], rice (*Oryza sativa* L.) [13], grape (*Vitis vinifera* L.) [14], wheat (*Triticum aestivum* L.) [15], radish (*Raphanus sativus* L.) [16], and tomato (*Solanum lycopersicum* L.) [17]. The *AtCPAs* reside in the plasma membrane, vacuoles, and chloroplasts, enabling ion exchange and transport, especially for monovalent cations in plants, including sodium and potassium ions [18, 19]. The *CPA* superfamily maintains cytoplasmic ion homeostasis and enhances salt tolerance through Na^+^ efflux and regionalization. NHX proteins have been reported in over 60 plant species [20]. The *Arabidopsis* NHX family comprises eight members (*AtNHX1*-*AtNHX8*) [21–23] involved in Na^+^ and H^+^ exchange and transport. *AtNHX1* was the first characterized NHX gene, significantly enhancing salt tolerance in transgenic yeast or plants [21, 24]. Overexpressing *AtNHX7* reduced Na^+^ accumulation in the xylem and stem, improving salt tolerance in transgenic *A. thaliana* [11]. The *NHX1* gene was reported to regulate salt tolerance in *Arachis hypogaea* and *Helianthus tuberosus* [25, 26]. Cloned from a drought-hardy legume, *VuNHX1* can also regulate salt tolerance [27]. In rice, *OsNHX1-5* and *OsNHX7*/*OsSOS1* have been identified to enhance salt tolerance [28, 29]. *OsCHX14* played a role in the K^+^ homeostasis process [30]. *AtKEA1-AtKEA3* played crucial roles in chloroplast osmoregulation and pH homeostasis [18], while *AtKEA2* regulated K^+^ and pH in plastids [31]. The CHX family proteins were involved in K^+^, Na^+^, and H^+^ transport. A total of 28 CHX genes were identified in *A. thaliana* [32]. *AtCHX13*, *AtCHX17*, *AtCHX20*, and *AtCHX23* predominantly transported K^+^ rather than Na^+^ [9]. *AtCHX14*, *AtCHX21*, *AtCHX23*, and *AtCHX24* played roles in K^+^ redistribution, salt tolerance, chloroplast development, pH homeostasis, and leaf senescence [33–36].

Potato (*Solanum tuberosum* L.) is the world’s third-largest food crop, valued for its rich nutrition, high yield, and adaptability. However, potato is relatively sensitive to salt stress. Excess salt in the soil reduces photosynthetic rates, disrupts ion balance, and impairs osmotic regulation, affecting tuber growth and development, leading to yield loss. Identifying genes conferring resistance to abiotic stress is crucial for molecular breeding in potato. Although some *CPA* genes have been linked to NaCl stress response in other plants, detailed information on *CPA* in potatoes remains limited. Our study aimed to systematically identify the *CPA* superfamily in the potato genome and analyze their phylogenetic relationship, gene structure, and conserved motifs. Additionally, we investigated the expression patterns of *StCPAs* under salt (NaCl) treatment and confirmed the sodium ion transport function of *StNHX1*, *StNHX2*, *StNHX3*, *StNHX5*, *StNHX6* and *StCHX19*. These findings lay the groundwork for further understanding of the role of *StCPA*s in NaCl stress responses.

## Results

### Identification and characterization of CPAs in potato

To identify CPA members in potato, we performed a BLAST search against the potato protein database using known CPA sequences from *Arabidopsis*. We identified 33 StCPA members in the potato genome, categorized into three subfamilies: StNHXs (*n* = 7), StCHXs (*n* = 20), and StKEAs (*n* = 6). The genes were named *StNHX1*-*StNHX7*, *StCHX1*-*StCHX20*, and *StKEA1*-*StKEA6* based on homologies with other species. Detailed information, such as gene name, coding region length, protein length, molecular weight, theoretical isoelectric point (pI), and subcellular localization predictions for all members, were analyzed (Table 1). Notably, all StCPAs contained a Na^+^/H^+^ exchange domain, with lengths of 281 to 1,828 amino acid and molecular weights of 30 to 206 kDa. The theoretical pI ranged from 4.48 to 9.76, indicating different protein charges. The subcellular localization of StCPA predicted by WoLF PSORT, and most of them were on the plasma membrane, in line with the function of maintaining Na^+^ homeostasis as transporters. In addition, StCPA are located on several organelles, including the endoplasmic reticulum, vacuole, cytoplasm, golgi apparatus and peroxisome.

**Table 1:** Physiochemical properties of *StCPA* genes

Gene ID	NCBI Accession	Family members	CDS	Amino acid	MW (kDa)	PI	Predicted subcellular location
DM8C01G00050	XP_006364070	StNHX1	3462	1154	127.86087	6.24	Plasma membrane
DM8C01G20540	XP_006342736	StNHX2	1614	538	59.45057	8.57	Plasma membrane
DM8C01G34890	XP_015164436	StNHX3	1611	537	58.81516	7.95	Plasma membrane / Vacuole
DM8C04G11110	XP_006364429	StNHX4	2898	966	106.17462	6.5	Plasma membrane
DM8C04G19620	XP_006350401	StNHX5	1650	550	60.89645	6.07	Plasma membrane
DM8C06G00790	XP_006362981	StNHX6	1605	535	59.05204	7.13	Plasma membrane
DM8C10G01190	XP_006352530	StNHX7	5484	1828	206.77168	7.45	Plasma membrane / Golgi apparatus
DM8C02G02680	XP_006361543	StCHX1	2442	814	89.81156	5.98	Plasma membrane
DM8C02G14740	XP_006355611	StCHX2	2445	815	88.22928	8.64	Chloroplast
DM8C03G03420	XP_006343341	StCHX3	2373	791	87.29633	6.38	Plasma membrane
DM8C04G10060	XP_027772185	StCHX4	2376	792	87.48286	7.85	Plasma membrane
DM8C05G22870	XP_006365323	StCHX5	843	281	30.41235	9.76	Cytoplasm / Plasma membrane
DM8C06G00430	XP_006355820	StCHX6	2424	808	89.3439	8.13	Cytoplasm / Plasma membrane
DM8C06G10320	XP_049412342	StCHX7	2499	833	91.61628	7.47	Plasma membrane
DM8C06G17510	XP_006366744	StCHX8	2526	842	91.99454	7.08	Plasma membrane
DM8C06G32020	XP_049408644	StCHX9	4026	1342	147.97606	8.63	Plasma membrane
DM8C08G04520	XP_049349907	StCHX10	2289	763	82.3556	7.96	Plasma membrane
DM8C08G04540	XP_015084067	StCHX11	1275	425	45.07818	9.5	Peroxisome
DM8C08G06830	XP_006366098	StCHX12	2499	833	91.88195	5.26	Plasma membrane
DM8C08G25290	XP_006355520	StCHX13	1881	627	68.64146	6.17	Plasma membrane / Cytoplasm
DM8C08G28710	XP_006343907	StCHX14	2409	803	87.00402	8.47	Plasma membrane / Cytoplasm
DM8C08G28720	XP_006343906	StCHX15	2409	803	86.62846	8.41	Plasma membrane
DM8C09G02150	XP_006341222	StCHX16	2391	797	87.69327	8.66	Plasma membrane
DM8C09G11130	XP_015161582	StCHX17	2370	790	87.81288	7.27	Plasma membrane
DM8C12G15520	XP_049372846	StCHX18	2334	778	85.90788	8.3	Plasma membrane
DM8C12G21940	XP_006360740	StCHX19	2382	794	86.34204	8.03	Plasma membrane / Endoplasmic reticulum
DM8C05G22880	XP_049366540	StCHX20	1335	445	49.95676	6.19	Cytoplasm
DM8C01G30690	XP_006339534	StKEA1	3606	1202	129.37718	4.71	Plasma membrane
DM8C03G16940	XP_006348050	StKEA2	1734	578	62.96448	7.55	Plasma membrane
DM8C05G13370	XP_049403596	StKEA3	1749	583	62.53565	5.29	Plasma membrane
DM8C07G11010	XP_049360980	StKEA4	1728	576	62.35339	5.3	Plasma membrane
DM8C08G13920	XP_006360984	StKEA5	1800	600	64.77197	7.76	Plasma membrane
DM8C11G16760	XP_006359366	StKEA6	2811	937	101.45054	4.48	Chloroplast / Mitochondrial

### Phylogenetic tree and chromosome localization analysis

**Fig. 1:**
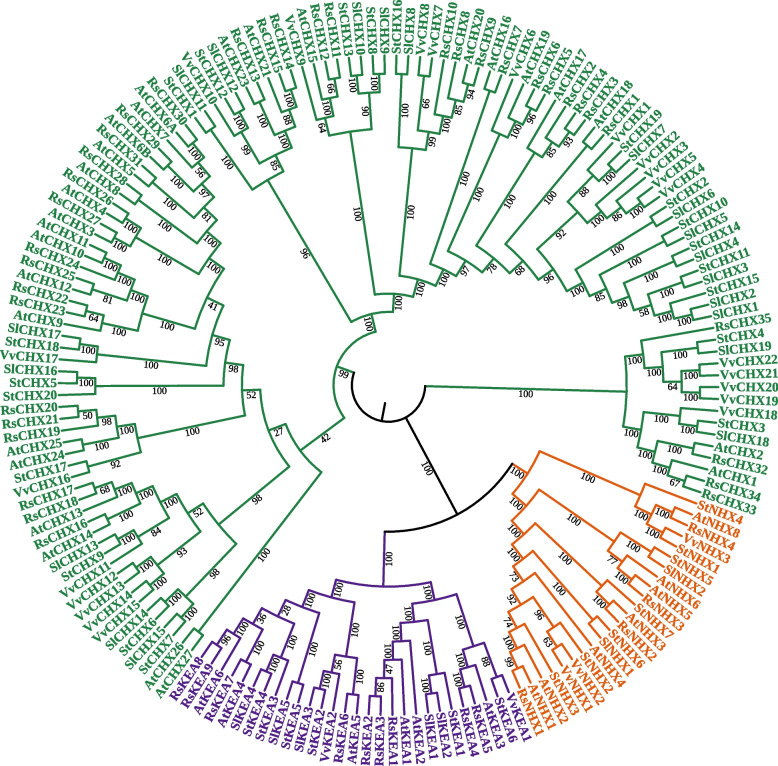
Phylogenetic tree of CPA proteins in potato, *Arabidopsis*, tomato, radish and grape. Neighbor-joining tree constructed using MEGA 11, depicting the phylogenetic relationship among 174 CPA proteins, comprising 40 AtCPAs, 33 StCPAs, 26 SlCPAs, 48 RsCPAs and 27 VvCPAs. The NHX, KEA, and CHX subfamilies encompass 23 members, 28 members, and 123 members, respectively

To analyze the phylogenetic relationships of all *StCPAs* members, we extracted full-length CPA protein sequences from potato, *Arabidopsis*, tomato, radish and grape, then aligned them to construct the neighbor-joining (NJ) phylogenetic tree (Fig. 1).

In total, 174 CPA proteins from five species (33 from potato, 40 from *Arabidopsis*, 26 from tomato, 48 from radish and 27 from grape) were categorized into three subfamilies: NHX, KEA, and CHX. Among them, the NHX group has 23 members, and the KEA group has 28 members. The CHX group is the most abundant subfamily, with 123 members, including 19, 22, 35, 27 and 20 from tomato, grape, radishe, *Arabidopsis* and potatoe, respectively. The phylogenetic tree indicated that the CPA gene family was highly conserved in different species.

**Fig. 2:**
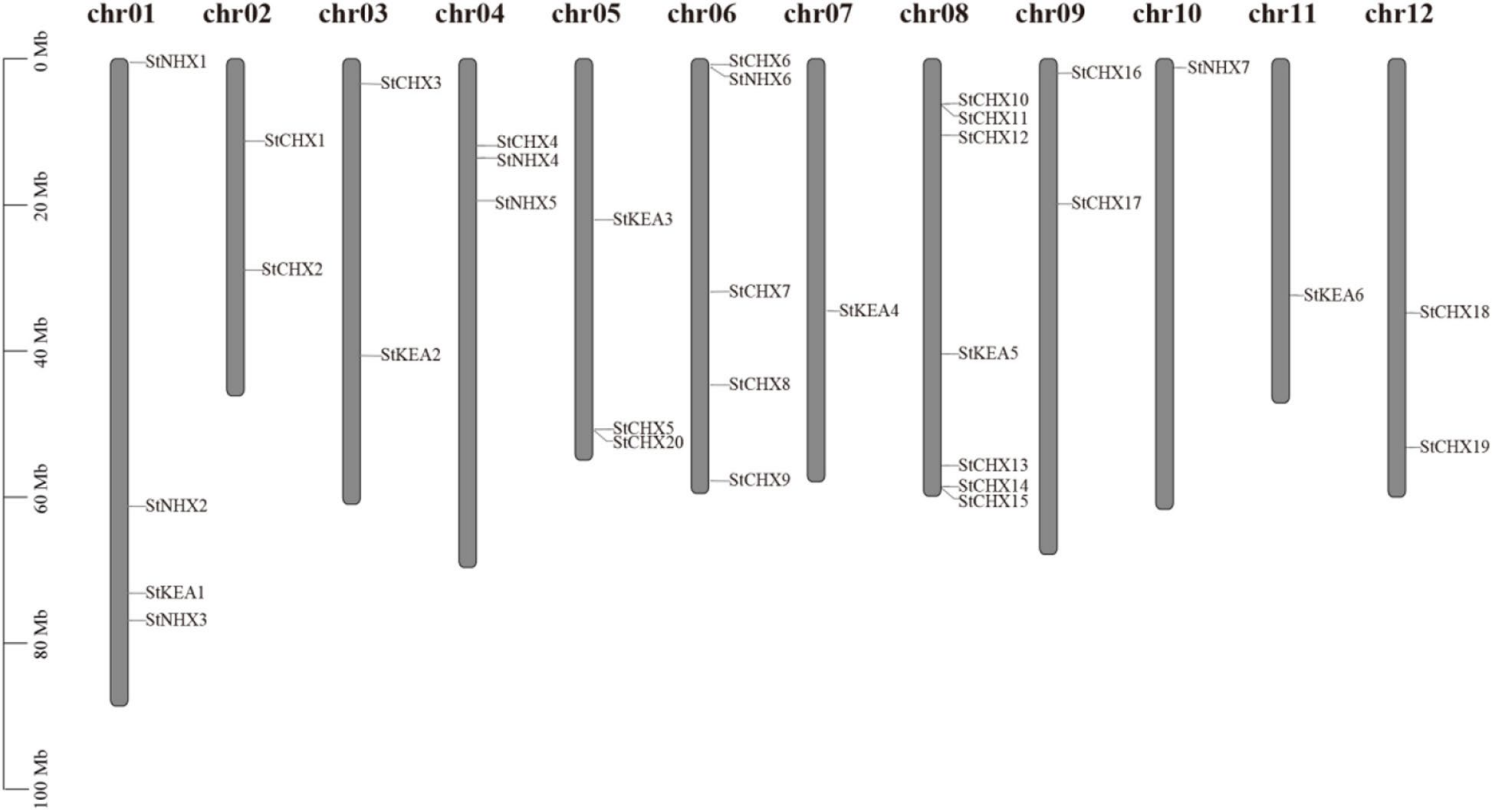
Chromosomal position of *StCPA* genes. Distribution of *StCPA* genes in potato chromosomes. Chromosome numbers are shown within each bar. The scale on the left is in megabases (Mb)

The *StCPA*s were mapped on the 12 chromosomes, and they unevenly distributed across different chromosomes, with one to seven genes on each chromosome (Fig. 2). Chromosome 8 had the most *CPA* members (seven), followed by five on chromosome 6 and four on chromosome 1. Chromosomes 4 and 5 each had three members, while chromosomes 2, 3, 9, and 12 each had two members. The remaining chromosomes each contained one member.

### Conserved motifs and gene structure analysis

**Fig. 3:**
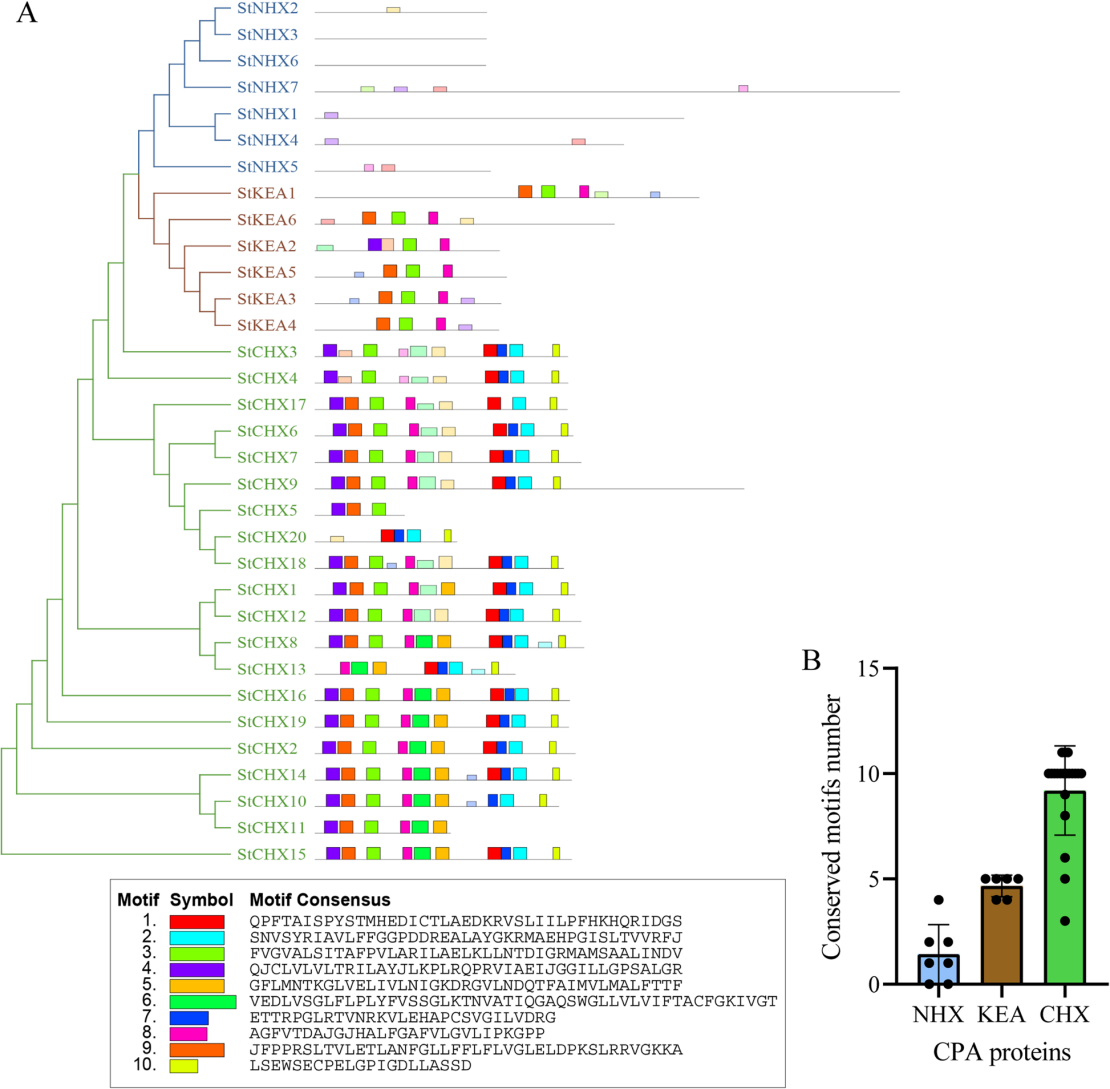
Conserved motifs of StCPA proteins. **A** Analysis of the conserved domains of the StCPA proteins. The 10 motifs are depicted using different color ranges. **B** Number of conserved motifs in StNHX, StKEA, and StCHX subfamilies

To distinguish the differences among the three *StCPA* families, we conducted an analysis of the gene composition and structure of the StCPA proteins (Fig. 3). StCHX members exhibited three to eleven conserved motifs, while KEA members had four to five conserved motifs. In the StNHXs family, StNHX3 and StNHX6 had no conserved motifs, StNHX1 and StNHX2 had only one conserved motif, StNHX4, and StNHX5 had two conserved motifs, and StNHX7 had four conserved motifs (Fig. 3A). Notably, most StCPA members within the same subfamily had similar motif compositions, implying conserved functions among these proteins. Additionally, the number of motifs varied across different subfamilies, with CHXs being the most abundant, followed by KEAs and NHXs (Fig. 3B). This suggests that the functions of different subfamilies may have evolved different functions over time.

**Fig. 4:**
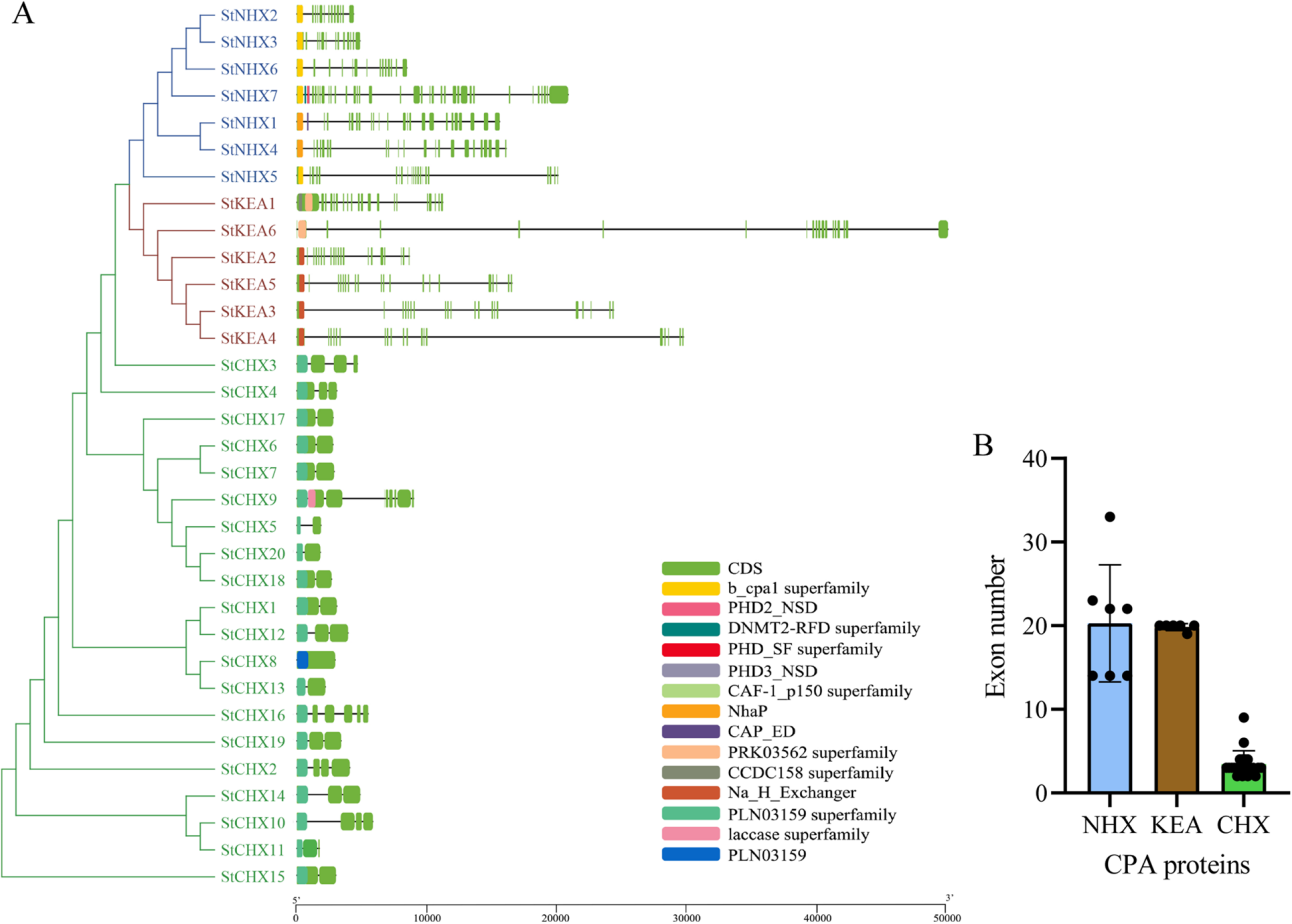
Structural analysis of the *StCPA* gene superfamily. **A** Structures of *StCPA* gene superfamily. Introns and exons are represented as thin lines and green boxes, respectively. **B** Number of exons in StNHX, StKEA, and StCHX subfamilies

We further analyzed the structural diversity of these *StCPA* (Fig. 4). The results revealed significant variations in sequence length and the number of introns/exons among *StCPA* members. The CHX family members had shorter sequence lengths and fewer exons, whereas the NHX and KEA family members had longer sequence lengths with many shorter exons (Fig. 4A and B). For instance, the *StKEA6* gene spans 46,476 bp and consists of 19 exons, while the *StCHX6* gene spans 2,596 bp and contains only 3 exons. This pattern is consistent with the *CPA* gene superfamily of tomato [17].

### RNA-seq analysis of the *CPA* gene superfamily under NaCl stress

**Fig. 5:**
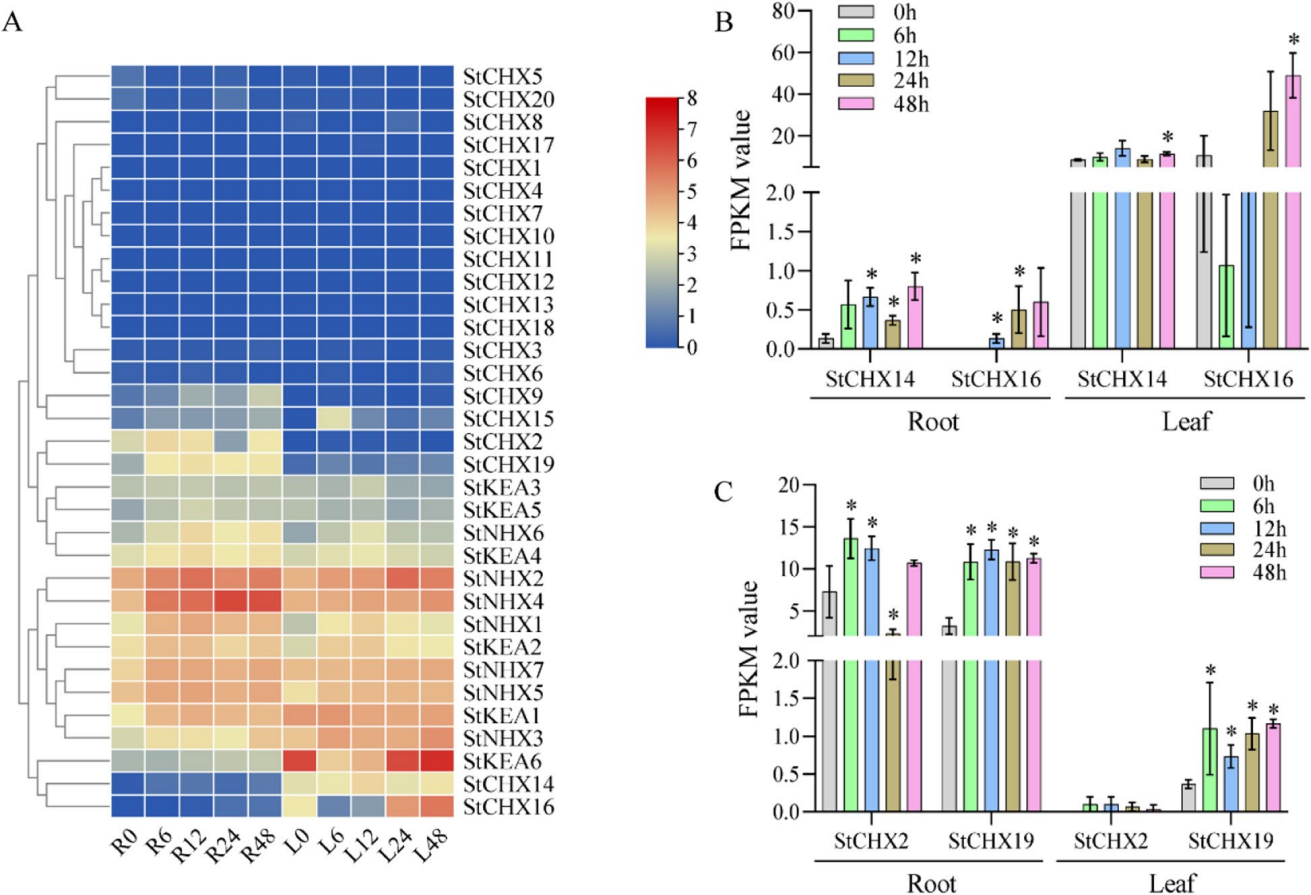
Expression of *StCPA* genes under NaCl treatment. **A** Heatmap of *CPA* superfamily RNA-Seq under NaCl stress. **B** Specific expression of genes *StCHX14* and *StCHX16* in leaves. **C** Specific expression of genes *StCHX2* and *StCHX19* in roots. Potatoes were subjected to 200 mM NaCl for 0 h, 6 h, 12 h, 24 h, and 48 h. Root samples (R0, R6, R12, R24, R48) and leaf samples (L0, L6, L12, L24, L48) were collected for RNA-Seq analysis. The asterisk indicates a significant difference (*p < 0.05)

To investigate the potential function of the *StCPA* superfamily under NaCl stress, we analyzed their expression patterns in leaves and roots under 200 mM NaCl treatment using RNA-seq data. The results revealed that the expression levels of 19 *StCPAs* were enhanced in roots or leaves under NaCl treatment (Fig. 5A). Notably, *StCHX14* and *StCHX16* exhibited specific high expression in leaves, while *StCHX2* and *StCHX19* are specifically expressed in roots, indicating that they may be specifically involved in regulating ion homeostasis in leaves or roots, thereby affecting NaCl tolerance (Fig. 5A and B). Interestingly, the remaining members (*n* = 14) of *StCHX* were not induced by NaCl stress, indicating that they may transport other cations but Na^+^. In contrast, all StNHX and StKEA were induced by NaCl stress, highlighting their potential roles in potato NaCl stress. Collectively, our findings suggest that St*CPA* members are differentially induced by NaCl stress and play distinct roles in NaCl resistance.

### Quantitative expression analysis of *StCPAs* under NaCl stress

**Fig. 6:**
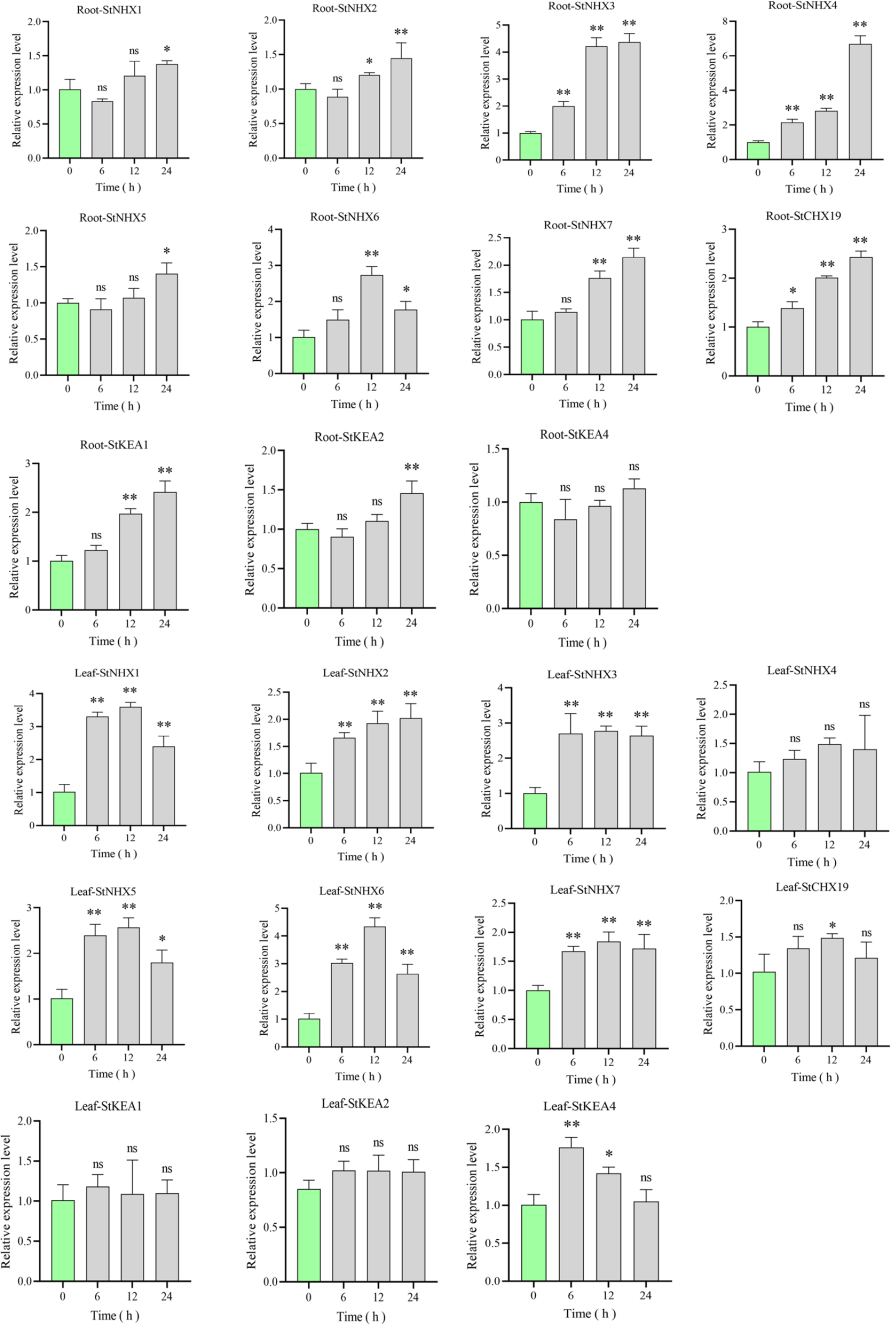
Quantitative RT-PCR expression analysis of the *CPA* gene superfamily. Expression profiles (qPCR) of the *StCPA* genes at different time intervals (0 h, 3 h, 6 h, 12 h, and 24 h) under 200 mM NaCl treatments. The relative expression levels at different stress treatment times were compared to the control (0 h). The asterisk indicates a significant difference (*p < 0.05;**p < 0.01); ns indicates the difference is not significant based on the t-test (*p* ≥ 0.05)

The expression of this family was further validated by qPCR analysis under NaCl stress (Fig. 6). We selected 11 *StCPA* genes (*StNHX1*, *StNHX2*, *StNHX3*, *StNHX4*, *StNHX5*, *StNHX6*, *StNHX7*, *StCHX19*, *StKEA1*, *StKEA2*, and *StKEA4*) that exhibited substantial induction by NaCl stress (Delta FPKM relative to CK > 10 in root or leaf). In roots, the expression level of *StNHX4* induced by NaCl stress is the highest (> 6-fold at the 24th hour). *StNHX3*, *StNHX6*, *StNHX7*, *StCHX19*, and *StKEA1* were induced to 2–3 folds. *StNHX1*, *StNHX2*, *StNHX5*, and *StKEA2* were significantly upregulated (*p* < 0.05), while the change of *StKEA4* was not significant. Notably, most of genes showed gradually increased expression with the prolongation of stress treatment, except for *StNHX6* with the highest upregulation at the12th hour. In leaves, the expression levels of *StNHX3* and *StNHX7* were quickly upregulated at the 6th hours (> 2-fold and > 1.5-fold) and remained this level; *StNHX1*, *StNHX5*, *StNHX6*, *StCHX19*, and *StKEA4* showed a trend of upregulation and then downregulation. Among them, *StNHX1*, *StNHX5*, *StNHX6*, and *StCHX19* showed the highest upregulation at the 12th hour and downregulation at the 24th hour, while *StKEA4* showed the highest upregulation at the 6th hours and gradually downregulated thereafter. The expression level of *StNHX2* was steadily upregulated in the leaves, while the other genes *StNHX4*, *StKEA1*, and *StKEA2* were not induced by NaCl stress in the leaves. In addition, we noticed that the induced expression levels of *StNHX3*, *StNHX4*, *StCHX19*, *StKEA1*, and *StKEA2* were higher in the roots than in the leaves, while the induced expression levels of *StNHX1*, *StNHX5*, *StNHX6*, and *StKEA4* in the leaves were higher in the roots. In summary, different *StCPA* members exhibited various responsive patterns, indicating their diverse functions in response to NaCl stress.

### Effect of NaCl stress on yeast cell growth

**Fig. 7:**
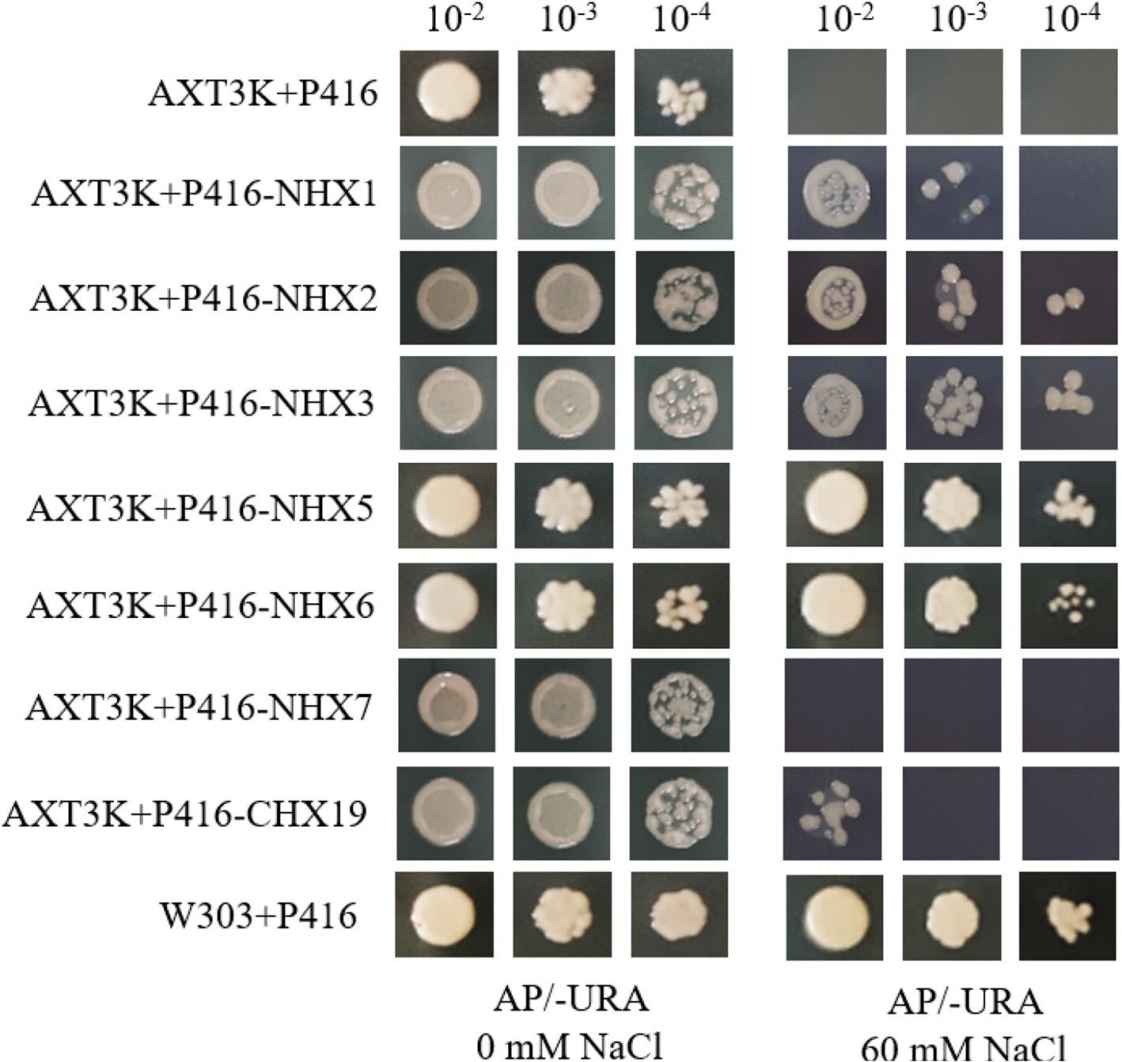
Effect of NaCl stress on yeast cell growth. The wild-type strain (W303 + P416), mutant cells (AXT3K + P416), and recombinant strains (AXT3K + P416-*StNHX1*, AXT3K + P416-*StNHX2*, AXT3K + P416-*StNHX3*, AXT3K + P416-*StNHX5*, AXT3K + P416-*StNHX6*, AXT3K + P416-*StNHX7*, and AXT3K + P416-*StCHX19*) were grown to saturation, and then 10-µL serial decimal dilutions were spotted onto AP-URA plates supplemented with 0 and 60 mM NaCl. Exogenous uracil was provided by the P416 vector to ensure normal growth of the strain in the AP-URA medium

In order to verify whether these responsive genes own the Na^+^ transport function, we selected seven genes, which strongly responded to NaCl stress both in roots and leaves, for validation using defective yeast expression experiments. These genes included *StNHX1*, *StNHX2*, *StNHX3*, *StNHX5*, *StNHX6*, *StNHX7*, and *StCHX19*. Under normal conditions, the growth of untransformed and transgenic strains is similar. Under NaCl stress, untransformed yeast cells AXT3K-P416 cannot grow on AP-URA medium, while the transgenic strains containing the functional genes could grow on AP-URA medium. The yeast strain that AXT3K + p416-*StNHX7* present the phenotype similar to AXT3K + P416 and cannot grow on AP-URA medium containing 60 mM NaCl, indicating that *StNHX7* does not have Na^+^ transport function. It is speculated that this gene is responsible for transporting cations other than Na^+^. In contrast, AXT3K strains transformed *StNHX1*, *StNHX2*, *StNHX3*, *StNHX5*, *StNHX6*, and *StCHX19* genes could grow in 60 mM NaCl AP-URA medium, indicating that these genes have Na^+^ transport functions. *StNHX5* and *StNHX6* showed the comparable phenotype with the positive control (W303 + P416), indicating their effective functions. The other members growth status was not as strong as the positive control W303 + P416, indicating that they only partially rescued the Na^+^ deficient phenotype of the AXT3K strain (Fig. 7). These results are consistent with previous research [15, 37].

## Discussion

In this study, we identified 33 CPA members in potato, classified into three subfamilies: CHX, NHX, and KEA. This classification was consistent with previous research on various plant species, including monocotyledons and dicotyledons like rice, maize, sorghum, *A. thaliana*, grape, and tomato [5, 11–17], This indicates that the evolution and classification of CPA proteins in different species is highly conserved. This study will provide reference for the subsequent functional characterization of the potato *CPA* gene family (Fig. 1). We observed significant differences in the number of conserved motifs among the subfamilies, with 0–4 motifs for StNHX, 4–5 motifs for StKEA, and 3–11 motifs for StCHX. Similar observations have been found in other species. For instance, in tomato, the number of motifs for SlNHX, SlKEA, and SlCHX is 0–2, 4–7, and 7–13, respectively [17]. In wheat, the number of motifs for TaNHX, TaKEA, and TaCHX is 1–4, 7–9, and 3–11, respectively [15]. In radish, the number of motifs for RsNHX, RsKEAs, and RsCHX is 1–4, 4–5, and 3–17, respectively [16]. Moreover, we also observed common features in the number of exons in different subfamilies across various species. For *StNHXs*, *StKEAs*, and *StCHXs*, the numbers of exons are 14–33, 19–20, and 2–9, respectively. Similarly, in tomato, the numbers are 1–23, 7–20, and 2–7 [17]. In wheat, the numbers are 7–25, 12–21, and 1–4 [15]. In radish, the numbers are 10–19, 17–21, and 1–5, respectively [16]. This consistency among different species indicates that *CPAs* are relatively conserved during evolution.

To date, numerous studies have reported the involvement of the *CPA* gene superfamily in NaCl tolerance in plants [9, 38]. Our qPCR results demonstrated that all 11 selected *StCPA* genes were upregulated in response to NaCl stress in leaves or roots, indicating that upregulating the expression of antiporters is an important mechanism for coping with NaCl stress. SOS1 (Salt overly sensitive 1) protein, a Na^+^/H^+^ antiporter found in various plants, is induced by NaCl stress and functions in Na^+^ efflux to enhance NaCl tolerance in plants [1, 3, 39]. In potato, *StNHX1* is the homologous gene of *AtSOS1*. We observed significant induction of *StNHX1* by NaCl stress in both leaves and roots of potato, consistent with our expectations. Furthermore, we noticed tissue-specific differences in gene expression. RNA-Seq showed that *StCHX14* and *StCHX16* were specifically expressed in leaves, while *StCHX2* and *StCHX19* were specifically expressed in roots. qPCR data showed that the expression level of *StNHX4* increased by 7 folds in the roots after 24 h of NaCl stress, while it was not significant in the leaves. This further proved that *StNHX4* play the major role in the roots. *StKEA4* showed a significant increase in expression levels, but not induced in the roots, suggesting that this gene mainly plays a role in the leaves. So, it is speculated that different members have different tissues-specific functional divergence of regulating NaCl tolerance.

In addition, we observed that the expression levels of all StNHX and most (5/6) StKEA members significantly increased in roots or leaves under NaCl stress. For StCHX subfamily, six members (*StCHX9*, *StCHX15*, *StCHX2*, *StCHX19*, *StCHX14*, and *StCHX16*) induced by NaCl stress, while most of (*n* = 14) members were not sensitive to NaCl stress. This indicated StNHX and StKEA play major roles in coping with NaCl stress, while StCHX subfamily members may be induced by various forms of salt stress.

The yeast system for rapid verification of *CPA* function has been widely employed in various plants [15, 40, 41]. For instance, heterologous expression of *ZmNHX5*, *ZmCHX2*, *ZmCHX3*, *ZmCHX5*, *ZmCHX17*, and *ZmKEA2* restored the Na^+^ resistance of the yeast mutant AXT3K, and the NaCl tolerance function of *ZmCHX2* and *ZmNHX5* was confirmed in transgenic plants [40]. Similarly, the wheat *CPA* gene superfamily *TaNHX4* enhanced the survival rate of *Escherichia coli* under various abiotic stresses [15]. In *Arabidopsis*, the *AtKEA* subfamily, except for *AtKEA3*, improved the tolerance of yeast mutant strains to high K^+^ stress [41]. To verified which member own ion transport function, a total of seven genes were applied to a yeast system. The results showed that heterologous expression of *StNHX1*, *StNHX2*, *StNHX3*, *StNHX5*, *StNHX6*, and *StCHX19* enhanced the NaCl tolerance of AXT3K, indicating that these genes indeed have potential Na^+^ efflux function. However, *StNHX7* did not rescue the defective expression of AXT3K, indicating that it does not have Na^+^ efflux function. It is speculated that this gene is induced to respond to NaCl stress and affects potato NaCl tolerance by transporting other metal ions.

## Conclusions

In this study, we conducted a comprehensive investigation of the *CPA* superfamily members in potato. A total of 33 *StCPA* genes were identified and categorized into three subfamilies: NHX, KEA, and CHX, with significant differences in conserved motifs and exons. More than half of the *StCPA* genes were induced by NaCl stress, exhibiting varying magnitudes and response times. Notably, *StNHX1*, *StNHX2*, *StNHX3*, *StNHX5*, *StNHX6*, and *StCHX19* were found to transport Na^+^ and enhance NaCl tolerance in defective yeast mutants. These findings provide valuable insights for future research on the biological functions and molecular mechanisms of these potato *CPA* genes in response to NaCl stress.

## Materials and methods

### Identification of the *CPA* gene superfamily in potato

The *Arabidopsis* CPA protein sequences were acquired from the TAIR database (https://www.arabidopsis.org/index.jsp) [42]; tomato CPA protein sequences were acquired from the phytozome database (https://phytozome-next.jgi.doe.gov/) [17]; radish CPA protein sequences were acquired from the National Genomics Data Center (NGDC) Genome Sequence Archive (GSA) (https://ngdc.cncb.ac.cn/gsa/) [43]; grape CPA protein sequences were acquired from the Genome Warehouse (GWH) database of the National Genomics Data Center (https://bigd.big.ac.cn/) (Supplementary Table S3) [44]. The potato genome and amino acid sequences were obtained from the National Genome Science Data Center (https://ngdc.cncb.ac.cn/) [45], BioProject: accession no. PRJCA011810. A BLAST-P search was conducted to query the StCPA proteins within the potato protein database, and the filtering threshold was established as an E-value less than e^−5^. These were further verified by interrogating the Pfam database (http://pfam.xfam.org/search/) [46] for the existence of a signature Na^+^/H^+^ exchanger (PF00999) domain. A total of 33 *StCPA* gene superfamily proteins were identified for subsequent analysis. Additionally, ExPASy ProtParam (https://www.expasy.org/) was employed to predict the physicochemical properties of StCPA proteins, including the length of the coding region, number of amino acids, molecular weight, theoretical isoelectric point (pI) [47]. Subcellular location of the StCPAs proteins predicted by WoLF PSORT (https://www.genscript.com/wolf-psort.html/) [40].

### Phylogenetic tree and chromosome localization analysis

Use MEGA11 software to align the CPA protein sequences of potato, *Arabidopsis*, tomato, radish, and grape, to construct a neighbor-joining (NJ) phylogenetic tree. The chromosomal positions of different *StCPA* genes were identified based on the National Genomics Data Center (https://ngdc.cncb.ac.cn/) [45], BioProject: accession no. PRJCA011810. The chromosomal map was visualized using the Ritchie-lab phenogram tool (http://visualization.ritchielab.org/phenograms/plot/).

### Conserved motifs and gene structure analysis

The potato *StCPA* gene superfamily sequence information was consistent with the above method. The structural information related to the *StCPA* genes was displayed using TBtools (https://github.com/CJ-Chen/TBtools/) [48]. The conserved motifs were identified by Multiple Em for Motif Elicitation (MEME) (http://meme.nbcr.net/meme/tools/meme/) [49].

### Planting and CPA gene superfamily RNA-Seq analysis

The potato genotype A056 was propagated using MS medium and cultured for 15 days in a growth room (conditions: light duration of 16 h, dark duration of 8 h; temperature of 21 ℃) and transplanted into a matrix block (the matrix block material is composed of coconut bran, peat, and wood, and after high-temperature sterilization, it was wrapped in a degradable mesh like non-woven fabric to form 41 mm × 42 mm sized matrix block). The seedlings were cultured for 2–3 days and grown normally over the course of 15 days. Add NaCl into tap water and prepare 200 mM NaCl aqueous solution and pour it into a seedling tray for potato seedlings to absorb and form NaCl stress. Leaf and root samples were collected from seedlings at 0 h, 6 h, 12 h, 24 h, and 48 h after NaCl treatment and flash-frozen in liquid nitrogen. Three biological replicates for each treatment were conducted. RNA was extracted using the TRIzol reagent method, and *StCPA* transcriptome analysis was performed. RNA-Seq was completed by the Beijing Annuo Company, China. The FPKM expression value was determined from the sequencing data and were further screened by heatmap drawing using TBtools software.

### Quantitative real-time PCR

The RNA samples for qPCR were identical to those used for RNA-Seq analysis. The first strand cDNA synthesis from total RNA reverse transcription was performed using the PrimeScript™ RT reagent Kit with gDNA Eraser (Perfect Real Time) from TaKaRa. Quantitative RT-PCR was performed using TB Green Premix Ex Taq II (Tli RNaseH Plus) (Code No. RR820A/B) from TaKaRa on a StepOnePlus Real-Time PCR Instrument (Applied Biosystems). Tubulin was employed as an internal control to normalize the samples. Primer design was conducted using Primer Premier 5 software and is outlined in Supplementary Table S1. All experiments were conducted with biological triplicates.

### Cloning of gene

Plant material accession A056 was utilized for total RNA extraction and reverse transcription polymerase chain reaction to generate cDNA sequences (an identical method to the described qPCR procedure). Primers were designed based on the CDS of *StNHX1*, *StNHX2*, *StNHX3*, *StNHX5*, *StNHX6*, *StNHX7*, and *StCHX19* from potato DM genomes. Detailed primer sequences can be found in Supplementary Table S2. In order to clone the coding region sequences of the *StNHX1*, *StNHX2*, *StNHX3*, *StNHX5*, *StNHX6*, *StNHX7*, and *StCHX19* genes, the cDNA of the A056 material was employed as a template for PCR amplification using the Supplementary Table S2 primers. The sequences of the *StNHX1*, *StNHX2*, *StNHX3*, *StNHX5*, *StNHX6*, *StNHX7*, and *StCHX19* genes were amplified and inserted into the P416 vector using the seamless cloning method (In-Fusion® Snap Assembly Master Mix) (TaKaRa). Finally, vector construction was verified by sequencing. The constructed vectors included P416-*StNHX1*, P416-*StNHX2*, P416-*StNHX3*, P416-*StNHX5*, P416-*StNHX6*, P416-*StNHX7*, and P416-*StCHX19*.

### Yeast strains and incubation

*Saccharomyces cerevisiae strain* AXT3K (△ena1::HIS3::△ena4,△nha1::LEU2, and △nhx1:: KanMX4), deficient in the main endogenous Na^+^ transporters, was used and is a derivative of W303 (MAT ura3-1 leu2-3,112 his3-11,15 trp1-1 ade2-1 can1-100) [37, 50]. To assess the functionality of the StNHX1, StNHX2, StNHX3, StNHX5, StNHX6, StNHX7, and StCHX19 proteins, p416-*StNHX1*, p416-*StNHX2*, p416-*StNHX3*, p416-*StNHX5*, p416-*StNHX6*, p416-*StNHX7*, and p416-*StCHX19* were transformed into AXT3K. Additionally, an empty P416 vector was transformed into W303 as a positive control and AXT3K as a negative control. The transformed strains were grown using an AP-URA medium for 72 h (AP culture medium comes from Shanghai Huzhen limited company, the instructions for use are detailed in the user manual). When precultures grew to saturation, they were diluted 100-fold, 1,000-fold, and 10,000-fold. Samples consisting of 2 µL of each series of diluents were dotted onto AP-URA plates containing 0 mM NaCl and 60 mM NaCl. After incubation for 3–5 days at 30 °C, growth was visualized and analyzed.

### Supplementary Information

**Additional file 1: Supplementary Table S1.** Primer sequences for qRT-PCR. **Supplementary Table S2.** Gene cloning primer. **Supplementary Table S3.** Arabidopsis, tomato, radish and grape CPA gene family information.

## Data Availability

The datasets generated and/or analyzed during the current study are available in the National Center for Biotechnology Information repository, (https://www.ncbi.nlm.nih.gov/sra/PRJNA1016159, accession number - PRJNA1016159).

## References

[1] Yang Y, Guo Y (2018). Elucidating the molecular mechanisms mediating plant salt-stress responses. New Phytol.

[2] Munns R, Tester M (2008). Mechanisms of salinity tolerance. Ann Rev Plant Biol.

[3] Yang Y, Guo Y (2018). Unraveling salt stress signaling in plants. J Integr Plant Biol.

[4] Gong Z, Xiong L, Shi H, Yang S, Herrera L, Xu G (2020). Plant abiotic stress response and nutrient use efficiency. Sci China Life Sci.

[5] Ye CY, Yang X, Xia X, Yin WL (2013). Comparative analysis of cation/proton antiporter superfamily in plants. Gene.

[6] Brett CL, Donowitz M, Rao R (2005). Evolutionary origins of eukaryotic sodium/proton exchangers. Am J Physiol-Cell Ph.

[7] Evans AR, Hall D, Pritchard J, Newbury HJ (2012). Retracted: The roles of the cation transporters CHX21 and CHX23 in the development of Arabidopsis thaliana. J Ex Bot.

[8] Pires IS, Negrao S, Pentony MM, Abreu IA, Oliveira MM, Purugganan MD (2013). Different evolutionary histories of two cation/proton exchanger gene families in plants. BMC Plant Biol.

[9] Chanroj S, Wang G, Venema K, Zhang MW, Delwiche CF, Sze H (2012). Conserved and diversified gene families of monovalent cation/H^+^ antiporters from algae to flowering plants. Front Plant Sci.

[10] Jia Q, Zheng C, Sun S, Amjad H, Liang K, Lin W (2018). The role of plant cation/proton antiporter gene family in salt tolerance. Biol Plant.

[11] Qiu QS, Guo Y, Dietrich MA, Zhu JK (2002). Regulation of SOS1, a plasma membrane Na^+^/H^+^ exchanger in *Arabidopsis thaliana*, by SOS2 and SOS3. P Natl A Sci USA.

[12] Cellier F, Conejero G, Ricaud L, Luu DT, Lepetit M, Gosti F (2004). Characterization of *AtCHX17*, a member of the cation/H^+^ exchangers, CHX family, from *Arabidopsis thaliana* suggests a role in K^+^ homeostasis. Plant J.

[13] Zeng Y, Li Q, Wang H, Zhang JL, Du J, Feng HM (2018). Two NHX-type transporters from *Helianthus tuberosus* improve the tolerance of rice to salinity and nutrient deficiency stress. Plant Biotechnol J.

[14] Ma Y, Wang J, Zhong Y, Cramer GR, Cheng ZM. Genome-wide analysis of the cation/proton antiporter (CPA) super family genes in grapevine (*Vitis vinifera* L.). Plant Omics. 2015;8:300–11.

[15] Sharma H, Taneja M, Upadhyay SK (2020). Identification, characterization and expression profiling of cation-proton antiporter superfamily in *Triticum aestivum* L. and functional analysis of *TaNHX4*-B. Genomics.

[16] Wang Y, Ying J, Zhang Y, Xu L, Zhang WT, Ni M (2020). Genome-wide identification and functional characterization of the cation proton antiporter (CPA) family related to salt stress response in radish (*Raphanus sativus* L). Int J Mol Sci.

[17] Hussain Z, Khan H, Imran M, Naeem MK, Shah SH, Iqbal A (2022). Cation/Proton antiporter genes in tomato: genomic characterization, expression profiling, and co-localization with salt stress-related QTLs. Agronomy.

[18] Kunz HH, Gierth M, Herdean A, Cruz MS, Kramer DM, Spetea C (2014). Plastidial transporters KEA1, 2, and-3 are essential for chloroplast osmoregulation, integrity, and pH regulation in *Arabidopsis*. P Natl A Sci USA.

[19] Han L, Li JL, Wang L, Shi WM, Su YH (2015). Identification and localized expression of putative K^+^/H^+^ antiporter genes in *Arabidopsis*. Acta Physiol Plant.

[20] Pardo JM, Cubero B, Leidi EO, Quintero FJ (2006). Alkali cation exchangers: roles in cellular homeostasis and stress tolerance. J Exp Bot.

[21] Apse MP, Aharon GS, Snedden WA, Blumwald E (1999). Salt tolerance conferred by overexpression of a vacuolar Na^+^/H^+^ antiport in *Arabidopsis*. Science.

[22] Apse MP, Sottosanto JB, Blumwald E (2003). Vacuolar cation/H^+^ exchange, ion homeostasis, and leaf development are altered in a T-DNA insertional mutant of *AtNHX1*, the *Arabidopsis* vacuolar Na^+^/H^+^ antiporter. Plant J.

[23] Yokoi S, Quintero FJ, Cubero B, Ruiz MT, Bressan RA, Hasegawa PM (2002). Differential expression and function of *Arabidopsis thaliana* NHX Na^+^/H^+^ antiporters in the salt stress response. Plant J.

[24] Gaxiola RA, Rao R, Sherman A, Grisafi P, Alper SL, Fink GR (1999). The *Arabidopsis thaliana* proton transporters, AtNhx1 and Avp1, can function in cation detoxification in yeast. P Natl A Sci USA.

[25] Zhang WW, Meng JJ, Xing JY, Yang S, Guo F, Li XG, Wan SB (2017). The K^+^/H^+^ antiporter *AhNHX1* improved tobacco tolerance to NaCl stress by enhancing K^+^ retention. J Plant Biol.

[26] Zeng Y, Li Q, Wang HY, Zhang JL, Du J, Feng HM, Blumwald E, Yu L, Xu GH (2017). Two NHX-type transporters from *Helianthus tuberosus* improve the tolerance of rice to salinity and nutrient deficiency stress. Plant Biotechnol J.

[27] Mishra S, Alavilli H, Lee B, Panda SK, Sahoo L (2015). Cloning and characterization of a novel vacuolar Na^+^/H^+^ antiporter gene (*VuNHX1*) from drought hardy legume, cowpea for salt tolerance. Plant Cell Tissue Organ.

[28] Fukuda A, Nakamura A, Hara N, Toki S, Tanaka Y (2011). Molecular and functional analyses of rice NHX-type Na^+^/H^+^ antiporter genes. Planta.

[29] Sellamuthu G, Jegadeeson V, Sajeevan RS, Rajakani R, Parthasarathy P, Raju K (2020). Distinct evolutionary origins of intron retention splicing events in *NHX1* antiporter transcripts relate to sequence specific distinctions in *Oryza* species. Front Plant Sci.

[30] Huanca-Mamani W, Ortiz MV, Cardenas-Ninasivincha S, Acosta-Garcia G, Bastias E (2018). Gene expression analysis in response to combined salt and boron (B) stresses in a tolerant maize landrace. Plant Omics.

[31] Aranda-Sicilia MN, Cagnac O, Chanroj S, Sze H, Rosales MPR, Venema K (2012). Arabidopsis KEA2, a homolog of bacterial KefC, encodes a K+/H+ antiporter with a chloroplast transit peptide. Biochim et Biophys acta (BBA)-biomembranes.

[32] Sze H, Padmanaban S, Cellier F, Honys D, Cheng NH, Bock KW (2004). Expression patterns of a novel *AtCHX* gene family highlight potential roles in osmotic adjustment and K^+^ homeostasis in pollen development. Plant Physiol.

[33] Song CP, Guo Y, Qiu Q, Lambert G, Galbraith DW, Jagendorf A (2004). A probable Na^+^ (K^+^)/H^+^ exchanger on the chloroplast envelope functions in pH homeostasis and chloroplast development in *Arabidopsis thaliana*. P Natl A Sci USA.

[34] Hall D, Evans AR, Newbury HJ, Pritchard J (2006). Functional analysis of CHX21: a putative sodium transporter in *Arabidopsis*. J Exp Bot.

[35] Hur Y, Kim JH, Lee DJ, Chung KM, Woo HR (2012). Overexpression of *AtCHX24*, a member of the cation/H^+^ exchangers, accelerates leaf senescence in *Arabidopsis thaliana*. Plant Sci.

[36] Zhao J, Li P, Motes CM, Park S, Hirschi KD (2015). CHX14 is a plasma membrane K-efflux transporter that regulates K^+^ redistribution in *Arabidopsis thaliana*. Plant Cell & Environ.

[37] Zhou Y, Yin X, Duan R, Hao GP, Guo JC, Jiang XY (2015). *SpAHA1* and *SpSOS1* coordinate in transgenic yeast to improve salt tolerance. PLoS ONE.

[38] Padmanaban S, Chanroj S, Kwak JM, Li XY, Ward JM, Sze H (2007). Participation of endomembrane cation/H^+^ exchanger AtCHX20 in osmoregulation of guard cells. Plant Physiol.

[39] Shi H, Ishitani M, Kim C, Zhu JK (2000). The *Arabidopsis thaliana* salt tolerance gene SOS1 encodes a putative Na^+^/H^+^ antiporter. P Natl A Sci USA.

[40] Kong M, Luo M, Li J, Feng Z, Zhang Y, Song W (2021). Genome-wide identification, characterization, and expression analysis of the monovalent cation-proton antiporter superfamily in maize, and functional analysis of its role in salt tolerance. Genomics.

[41] Zheng S, Pan T, Fan L, Qiu Q (2013). A novel AtKEA gene family, homolog of bacterial K^+^/H^+^ antiporters, plays potential roles in K^+^ homeostasis and osmotic adjustment in *Arabidopsis*. PLoS One.

[42] Lamesch P, Berardini TZ, Li D, Swarbreck D, Wilks C, Sasidharan R (2012). The *Arabidopsis* Information Resource (TAIR): improved gene annotation and new tools. Nucleic Acids Res.

[43] Xu L, Wang Y, Dong JH, Zhang W, Tang MJ, Zhang WL (2023). A chromosome-level genome assembly of radish (Raphanus sativus L.) reveals insights into genome adaptation and differential bolting regulation. Plant Biotechnol J.

[44] Shi XY, Cao S, Wang X, Huang SY, Wang Y, Liu Z (2023). The complete reference genome for grapevine (*Vitis vinifera* L.) genetics and breeding. Hortic Res.

[45] Yang X, Zhang L, Guo X, Xu JF, Zhang K, Yang YQ (2023). The gap-free potato genome assembly reveals large tandem gene clusters of agronomical importance in highly repeated genomic regions. Mol Plant.

[46] Finn RD, Bateman A, Clements J, Coggill P, Eberhardt RY, Eddy SR (2014). Pfam: the protein families database. Nucleic Acids Res.

[47] Gasteiger E, Gattiker A, Hoogland C, Lvan L, Appel RD, Bairoch A (2003). ExPASy: the proteomics server for in-depth protein knowledge and analysis. Nucleic Acids R.

[48] Chen C, Chen H, Zhang Y, Thomas HR, Frank MH, He YH (2020). TBtools: an integrative toolkit developed for interactive analyses of big biological data. Mol Plant.

[49] Bailey TL (2011). MEME-ChIP: Motif analysis of large DNA datasets. Bioinformatics.

[50] Quintero FJ, Ohta M, Shi H, Pardo JM (2002). Reconstitution in yeast of the *Arabidopsis* SOS signaling pathway for Na^+^ homeostasis. P Natl A Sci USA.

