# Supplementary material for: Genome-wide characterization of the CPA gene family in potato and a preliminary functional analysis of its role in NaCl tolerance
Source: BMC Genomics. 2024 Feb 5;25:144. doi: 10.1186/s12864-024-10000-2 (PMC10840148; doi:10.1186/s12864-024-10000-2)
Supplement: Supplementary file 1 — Additional file 1: Supplementary Table S1. Primer sequences for qRT-PCR. Supplementary Table S2. Gene cloning primer. Supplementary Table S3. Arabidopsis, tomato, radish and grape CPA gene family information. [file 12864_2024_10000_MOESM1_ESM.docx]

**Supplementary Table S1 Primer sequences for qRT-PCR**

| Gene Name | NCBI accession | Primer Name | Oligonucleotide Sequence (5’-3’) |
| --- | --- | --- | --- |
| StNHX1 | XP_006364070 | StNHX1-qRT-F  StNHX1-qRT-R | GCAGCCAAAACCTCTCACAG  GCCACCAGAATCATCACTCG |
| StNHX2 | XP_006342736 | StNHX2-qRT-F  StNHX2-qRT-R | CTCAAGAGTCACCACCAAGCA  CAACCAAAACAAGACCCAACA |
| StNHX4 | XP_006364429 | StNHX4-qRT-F  StNHX4-qRT-R | GGCGACAGTGGAGGAAGGT  CGAGAGAGCCGAGAGCGAT |
| StNHX5 | XP_006350401 | StNHX5-qRT-F  StNHX5-qRT-R | GTGTCGGCGTTTTTTCATTTG  TTTGCTGGTATCTTTTGGTGTG |
| StNHX6 | XP_006362981 | StNHX6-qRT-F  StNHX6-qRT-R | GCAGGTGCTTAATCAGGACGA  CGCACTTAGCAATCCAATCAA |
| StKEA2 | XP_006348050 | StKEA2-qRT-F  StKEA2-qRT-R | ATGTCATCAACGGCAGTGGT  TCAGCAGCACTTTTCCCATAG |
| StKEA4  StCHX19  StNHX7  StNHX3  StKEA1  EF1a | XP_049360980  XP_006360740  XP_006352530  XP_015164436  XP_006339534  XM_006343393 | StKEA4-qRT-F  StKEA4-qRT-R  StCHX19-qRT-F  StCHX19-qRT-R  StNHX7-qRT-F  StNHX7-qRT-R  StNHX3-qRT-F  StNHX3-qRT-R  StKEA1-qRT-F  StKEA1-qRT-R  StEF1a-F  StEF1a-R | GTGGGATTTCCTGTCTGTTTTC  ATTGCAGCCTTATTGGTGTAGC  TGCGTCAGAGTGGGAAAAAA  CAAAATGCGTGCCAGAACAG  CAGCAGGGTGATGGGTATTTG  ATTGTTGCACAGGCGTATTGA  CAAGAGTCACTACAAGGCACGC  TTGCTCCGAGGATTTCTTCAT  TAAAGATCGGGAGGTACAACTGG  CACTAAGCGGTGAACTGTCCC  GATGGTCAGACCCGTGAACA  CCTTGGAGTACTTCGGGGTG |

**Supplementary Table S2 Gene cloning primer**

| Primer Name | Oligonucleotide Sequence (5’-3’) |
| --- | --- |
| P416-CDS-StNHX1-F | ATGGAGTTGTTAGTAGAAACGAGGC |
| P416-CDS-StNHX1-R | CTATGATGTTTTATCTTCCTCTGTGAT |
| P416-CDS-StNHX2-F | ATGGTGTTTGACTTTGGGACG |
| P416-CDS-StNHX2-R | TTAATGACCACTTGGTTCAGTTGG |
| P416-CDS-StNHX3-F | ATGGCTTCTGTGCTGGCTTC |
| P416-CDS-StNHX3-R | TCATGGACCCTGTTCCGTTG |
| P416-CDS-StNHX5-F | ATGGAGGATCATCTTCAGATTTCT |
| P416-CDS-StNHX5-R | TCAGTGGCCGTCATAACCG |
| P416-CDS-StNHX6-F | ATGGGGTTGGATGCTGTGG |
| P416-CDS-StNHX6-R | TCAATATTGATCACATCCTCCCTT |
| P416-CDS-StNHX7-F | ATGGCCCTTTTCAATTACGTG |
| P416-CDS-StNHX7-R | TTACTCATTAAGCCACCCTGACG |
| P416-CDS-StCHX19-F | ATGGATCATCAGTGTCCATCTCC |
| P416-CDS-StCHX19-R | TTAATTCTCTGGAACTGATGCTACA |

**Supplementary Table S3 Arabidopsis, tomato, radish and grape *CPA* gene family information**

| Gene names | NCBI accession | Gene names | NCBI accession |
| --- | --- | --- | --- |
| AtNHX1 | NP_198067 | VvCHX1 | XP_002272080 |
| AtNHX2 | NP_001319475 | VvCHX2 | XP_002276249 |
| AtNHX3 | NP_200358 | VvCHX3 | WJZ80644 |
| AtNHX4 | NP_187288 | VvCHX4 | WJZ81937 |
| AtNHX5 | NP_178079 | VvCHX5 | WJZ81938 |
| AtNHX6 | NP_175839 | VvCHX6 | XP_002270854 |
| AtNHX8 | NP_172918 | VvCHX7 | XP_002262680 |
| AtKEA1 | NP_001320631 | VvCHX8 | XP_002269591 |
| AtKEA2 | NP_001190645 | VvCHX9 | XP_002262677 |
| AtKEA3 | NP_001190675 | VvCHX10 | WJZ96714 |
| AtKEA4 | NP_849990 | VvCHX11 | XP_002277552 |
| AtKEA5 | NP_568763 | VvCHX12 | XP_010650847 |
| AtKEA6 | NP_196741 | VvCHX13 | WJZ89181 |
| AtCHX1 | NP_173088 | VvCHX14 | XP_003632597 |
| AtCHX2 | NP_178058 | VvCHX15 | WJZ92891 |
| AtCHX3 | NP_197681 | VvCHX16 | WJZ89164 |
| AtCHX4 | NP_190076 | VvCHX17 | WJZ89178 |
| AtCHX5 | NP_172294 | VvCHX18 | XP_010651630 |
| AtCHX6A | NP_849611 | VvCHX19 | XP_059593710 |
| AtCHX6B | NP_849610 | VvCHX20 | WJZ91562 |
| AtCHX7 | NP_180384 | VvCHX21 | RVX02953 |
| AtCHX8 | KAG7637732 | VvCHX22 | WKA02370 |
| AtCHX9 | NP_197682 | RsNHX1 | XP_018457186 |
| AtCHX10 | NP_190079 | RsNHX2 | XP_018454884 |
| AtCHX11 | NP_190078 | RsNHX3 | XP_018442376 |
| AtCHX12 | NP_190077 | RsNHX4 | XP_018438776 |
| AtCHX13 | NP_180583 | RsKEA1 | XP_018490809 |
| AtCHX14 | CAD5311960 | RsKEA2 | XP_018487709 |
| AtCHX15 | NP_178985 | RsKEA3 | XP_018487697 |
| AtCHX16 | NP_176599 | RsKEA4 | XP_018452278 |
| AtCHX17 | NP_001328705 | RsKEA5 | XP_018452278 |
| AtCHX18 | NP_001332436 | RsKEA6 | XP_018443775 |
| AtCHX19 | NP_001319577 | RsKEA7 | XP_018441390 |
| AtCHX20 | NP_190940 | RsKEA8 | XP_018471132 |
| AtCHX21 | NP_180750 | RsKEA9 | XP_018444990 |
| AtCHX23 | NP_172049 | RsCHX1 | XP_018478140 |
| AtCHX24 | NP_198522 | RsCHX2 | XP_018448139 |
| AtCHX25 | NP_200654 | RsCHX3 | XP_018470344 |
| AtCHX26 | NP_195788 | RsCHX4 | XP_018483002 |
| AtCHX27 | KAG7600814 | RsCHX5 | XP_018489107 |
| SlNHX1 | XP_010321693 | RsCHX6 | XP_056854718 |
| SlNHX2 | NP_001315563 | RsCHX7 | XP_018456459 |
| SlKEA1 | XP_015056111 | RsCHX8 | XP_018489688 |
| SlKEA2 | XP_004229880 | RsCHX9 | XP_018478504 |
| SlKEA3 | NP_001307278 | RsCHX10 | XP_018442408 |
| SlKEA4 | XP_004242967 | RsCHX11 | XP_018435404 |
| SlKEA5 | XP_004245107 | RsCHX12 | XP_018455790 |
| SlCHX1 | XP_004245551 | RsCHX13 | XP_018434647 |
| SlCHX2 | XP_004245551 | RsCHX14 | XP_018482340 |
| SlCHX3 | TMW93660 | RsCHX15 | KAJ4903398 |
| SlCHX4 | XP_004245552 | RsCHX16 | XP_056847860 |
| SlCHX5 | XP_004244724 | RsCHX17 | XP_056861635 |
| SlCHX6 | XP_004233089 | RsCHX18 | XP_018482241 |
| SlCHX7 | XP_004252574 | RsCHX19 | XP_056858662 |
| SlCHX8 | XP_004246412 | RsCHX20 | XP_018474224 |
| SlCHX9 | XP_025887319 | RsCHX21 | XP_018455615 |
| SlCHX10 | XP_004246149 | RsCHX22 | XP_018444895 |
| SlCHX11 | XP_004231538 | RsCHX23 | XP_018444895 |
| SlCHX12 | XP_004244795 | RsCHX24 | XP_018487984 |
| SlCHX13 | XP_004242551 | RsCHX25 | XP_018438493 |
| SlCHX14 | XP_004240555 | RsCHX26 | XP_018469369 |
| SlCHX15 | XP_004241108 | RsCHX27 | XP_018469778 |
| SlCHX16 | XP_004239962 | RsCHX28 | KAG2308443 |
| SlCHX17 | XP_004252425 | RsCHX29 | XP_018471706 |
| SlCHX18 | XP_004234519 | RsCHX30 | XP_018471740 |
| SlCHX19 | XP_025886412 | RsCHX31 | XP_056841772 |
| VvNHX1 | XP_010649604 | RsCHX32 | KAJ4874418 |
| VvNHX2 | WJZ90259 | RsCHX33 | XP_018470454 |
| VvNHX3 | XP_002276346 | RsCHX34 | XP_018450208 |
| VvKEA1 | XP_059591960 | RsCHX35 | KAJ4873986 |
| VvKEA2 | XP_002269354 |  |  |
